# Perception of neighborhood environment and health risk behaviors in Prague’s teenagers: a pilot study in a post-communist city

**DOI:** 10.1186/1476-072X-13-41

**Published:** 2014-10-14

**Authors:** Jana Spilkova, Dagmar Dzúrova, Michal Pitonak

**Affiliations:** Faculty of Science, Department of Social Geography and Regional Development, Charles University, Albertov 6, Prague, 2 128 43 Czech Republic

## Abstract

**Background:**

A youths’ neighborhood can play an important role in their physical, health, and emotional development. The prevalence of health risk behavior (HRB) in Czech youth such as smoking, drug and alcohol use is the highest in Europe.

**Aim:**

To analyze differences in HRB in youth residents within different types of Prague’s neighborhoods in relation to the perception of the built environment, quality of their school and home environments.

**Data and methods:**

The data is based on the on-line survey among elementary school students aged between 14–15 years, which was administered in19 selected schools in Prague, during the months of October 2013 to March 2014. Respondents were asked their opinions on various issues related to their HRB, about their indoor and outdoor housing and school environments. The questionnaire was completed by 407 students. Factor analysis with a principal components extraction was applied to determine the underlying structure in the variables. A consequent field research was conducted to map the opportunity hot spots and critical places around the elementary schools.

**Results:**

Binge drinking has been reported mainly by the students living in the housing estates with blocks of flats. The most frequent occurrence of daily smokers was found in the neighborhoods of old city apartment houses. High prevalence of risky marijuana use almost in all the surveyed types of neighborhoods. The respondents were more critical in their evaluation of school characteristics. The neighborhoods critically evaluated by the students as regards the school outdoor environments were the older apartment houses in the historical centre and inner city, the school indoor environment was worst assessed within the housing estate neighborhoods.

**Conclusions:**

Our results suggest that perceptions of problems in both residential and school environment are associated with HRB. This fact makes this issue of a serious importance also from the policy point of view. Mainly the school surroundings have to be better managed by the local authorities responsible for the public space. This research thus forms part of the Sophie project aiming to find the most efficient policies that would tackle with the inequalities in the health and quality of life.

## Background

In the literature today, the influence of the environment on health problems is being approached from different points of view – of psychology, medicine, environmental sciences, social geography, social epidemiology, public health policies, etc. There is a growing interest in studying the relation between the built environment and health [1–3], with a strong suggestion that neighborhood environment characteristics play a role in the health and health behavior of residents, especially in relation to substance abuse and its consequences.

Weich et al. [4] showed that people in worse built environments demonstrated higher levels of resident depression. At the community level, certain characteristics of the neighborhoods that people inhabit have also proved to have harmful effects on health. These characteristics often include class, income or the racial structure of the community, but most of the studies also allow crime rates, threat of crime, local problems, physical hazards, noise, pollution etc. to enter the picture of environmental effects on health. These characteristics were earlier conceptualized into so-called *high-stress neighborhoods.* Harburg et al. [5] define these areas as places with low socioeconomic status, high population density, high geographic mobility, high rates of marital breakup, high crime, high stress etc.

Groves and Sampson [6] have then created the notion of *neighborhood disorganization*. Various authors consider many different characteristics in the depiction of this phenomenon: Crum et al. [7] include the perception of walkable areas or playgrounds, safety, crime, racism, litter, vandalism, visible public alcohol or drug use, abandoned buildings etc. Others [8, 9] also add graffiti, noise and dirt. Bernstein et al. [10] note that it is not only the buildings in a neighborhood that should be assessed, as streets, parks, playgrounds, public spaces etc. also have a potential influence on health. In relation to disorganization theories, ecological research also posits that some places have features that facilitate opportunities for risk behavior. These opportunity theories in general explain variations in crime or other pathological phenomena as due to the physical environment, predisposed structural dynamics (thus the social disorganization) of neighborhoods and victim lifestyles [11].

Another community characteristic with an obvious adverse effect on health is the absence of social networks and social ties. The social environment is important because it forms the social norms in the neighborhood and enforces the mechanisms of social control [12]. However, strong *social capital* cannot be created within unstable communities. Distrust of others causes social isolation, which in turn relates to other detrimental health outcomes [13]. High levels of social isolation also create a barrier to collaboration and to joint efforts to reduce the diverse signs of neighborhood disorder [14]. In connection with social capital and stable communities, the lifestyle theory [15] states that different social groups have different lifestyles with varying exposure to dangerous places, times and other individuals. According to this theory the place of socializing and time spent outside of the home (at school in our case) should be examined.

These aspects of neighborhood disorder are often studied in relation to adolescent risk behavior [16–18]. In this sense, many studies also insist that not only aspects of neighborhoods, but also school environments have to be taken into account [19, 20]. When it comes to adolescent risk behavior, the school environment, school norms, peer affiliation and social bonding are associated with adolescent drug use [21, 22].

It is obvious that the study of adolescent risk behavior in the context of the community environment is a highly complex domain which has to take into account many aspects ranging from the built environment, social environment and community cohesion through the school setting and peer influence to family background and the mental health moderation of a person’s behavior choices.

However, research into neighborhood disorganization and health risks yields mixed results. The majority of the work on environment and risk behavior suggests that neighborhood disorder is associated with a loss of social control and thus a higher risk of substance use [23], drug use as a coping mechanism [24], higher consumption of alcohol [9] and vice versa (thus high social cohesion and satisfaction with the neighborhood imply lower alcohol and drug use among adolescents [25, 26, 18]). However, there are also numerous studies showing that high income neighborhoods may have increased reporting of parental drinking, which further influences adolescent alcohol use [27], or that affluent neighbors can have undesirable effects through relative deprivation, cultural conflict etc. [28].

This implies that more types of environment have to be examined, both disorganized and affluent ones. At the same time, the focus should shift to the place as the unit of analysis at the micro-environmental level (block, block groups within neighborhoods), which provides the necessary level of detail to capture variations in the independent variables related to risk behavior [29]. As Gottfredson [30] notes, large-scale and aggregate surveys fail to distinguish the characteristics and features of particular areas that may be associated with greater risks. What is more, all the complex spectrum of variables have to be taken into account in multilevel analyses of the relationship between environment and health risk behavior.

This is especially true in the post-communist context, which exhibits many specific features. First of all, the influence of the neighborhood is not as accentuated as in U.S. studies, where racial, socioeconomic, or even religious heterogeneity tends to determine or strongly correlate with the quality of neighborhoods. The long period of equalization within communist societies erased social inequalities, which began to emerge only after the reestablishment of market principles. The housing estates with concrete blocks of flats, the typical built environment of the communist era, housed a wide spectrum of resident classes from manual or blue collar workers to the intelligentsia and elites (university professors, lawyers etc.). Even today, due to the lack of affordable housing in Prague, these estates accommodate a socio-economic mixture of inhabitants. The same is true for suburbia (former villages around the metropolitan area), which accommodate both the “new rich” in expensive gated communities of luxurious family houses and the former dwellers in traditional family houses with a different socio-economic profile. Secondly, crime or delinquency issues tend to differ, as there are different opportunities and mechanisms for e.g. the purchase of drugs, drug dealing, and possession of weapons etc., so variables more appropriate for the given context have to be chosen for the evaluation of the neighborhood environment. Third, previous studies also showed that the post-communist countries (primarily Czechia) are societies that are widely tolerant to risk behavior, enabling easy access to alcohol, cigarettes and illicit drugs for young people as well as low social control at the level of communities. It is thus extremely important to provide a pilot survey aimed at identifying the relation between the built environment and risk behavior of teenagers based on variables relevant to the context of a post-communist city. In our paper we introduce the case of Prague, the capital of Czechia, where a pilot study on built environment and health risk behavior has been conducted taking into account home and school environments in different types of neighborhoods. We are not aware of any other similar study that takes into consideration the health risks and built environment conducted in Czechia or other post-communist countries.

### The case of Prague’s teenagers

In the Czech Republic, as in other countries of Central and Eastern Europe, the political changes during the transition period brought changes in the life style and behaviors of many citizens, including higher alcohol consumption, drug use, and cigarette smoking [31]. Czech society is however traditionally rather tolerant of regular drinking of alcohol as well as excessive drinking. These tolerant norms may create similar attitudes to alcohol among children and young people. The price of alcohol is relatively low when compared with Western Europe, making alcohol more accessible and influencing the negative effects of (mis)use. This seems to be confirmed by the results of the European School Survey Project on Alcohol and Other Drugs 2011 (ESPAD) which showed that 93% of Czech teenagers aged 15–16 used alcohol during the 12 months prior to the survey and 79% during the previous month. Similarly, smoking is a substantial problem among young people with 43% of teenagers smoking during the month before the survey (with a slightly higher prevalence among girls). The same is true for drug use, because some of the illicit drugs have become “fashion” items for many young people. For example, according to the ESPAD report, the lifetime prevalence of cannabis use among Czech adolescents (42%) is significantly higher than in the rest of countries selected (the European average is 17%). Czech results also demonstrate the highest perceived availability of cannabis (59%) which is twice the European average [32].

Since Prague is the capital city of Czechia with more than one and half million inhabitants, it can be expected that the negative outcomes of the above mentioned trends will be even more prevalent here. Several studies [33, 34] also suggest that with the increasing size of the settlement, the odds ratios of particular health risk behaviors among teenagers (smoking, alcohol drinking and marijuana use) increase significantly. Therefore, the situation may be compared to the trends in developed countries as described in the background section. On the other hand, the structure and dynamics within a post-communist city vary greatly as a consequence of the different transformation processes, historical context and socio-economic development within a particular country. According to Enyedi [35], the combination of local political and economic transition postponed the shift from the industrial to the post-industrial phase in Central European countries. This, in combination with a general transformation of the global economy, creates the unique conditions of urban development in the post-communist heart of Europe.

The aim of this paper is to present a first study of the effects of the built environment on the health risk behavior of Prague teenagers. The methodology of the survey is based on the idea of the mixed method approach, defined as a research strategy involving more than one type of research method, which is among the most promoted research strategies nowadays [36]. It applies the fusion of quantitative and qualitative techniques in a different sequence (e.g. primary mapping of the environment, a stage of participant observation, collection of photographic materials, field research notes, carrying out a questionnaire survey, statistical data analysis, the addition of structured in-depth interviews, etc.). The advantage of the mixed method approach consists in the balancing of the strong and weak aspects of qualitative and quantitative research, the possibility of obtaining more complex results and the cooperation of specialists with different methodological backgrounds. Among the disadvantages, the higher requirements for expertise and resources are often cited.

The plan of our research was divided into two phases: a quantitative and a qualitative one.

1. In a pilot study using a questionnaire survey, we first focused on the students’ subjective perceptions of both outdoor and indoor features characterizing the quality of their home and school environment and on their reported health risk behavior. The principal research questions were:
  Does the quality of home and school environment influence the health risk behaviors of teenagers living in different built environment neighborhood types? Is there a different perception of home and school environments by teenagers living in different built environments?
2. In the second phase, field research was conducted, documenting the particular neighborhood characteristics of the built environment around the schools chosen for the questionnaire survey. The critical points were identified and mapped in situ and photographical evidence of these hot spots was collected. The main questions of the qualitative phase were:
  Do the “problematic” and “less problematic” neighborhoods differ in the range of opportunities for risk behavior as regards the physical environment, the predisposed structural dynamics of the neighborhood and routine activities of the teenagers? What are the characteristics of the problematic environments that should be first targeted by public health policies?

### Data and methods: the questionnaire survey of Prague’s teenagers

We use the data collected through an on-line survey among elementary school students, which was administered in 19 selected schools in Prague, from October 2014 to March 2014. The schools were selected according to their neighborhood type so that they represented different built environments. The seven built environments included blocks of flats in housing estates, new family houses in the suburbia, row houses, old city apartment houses, newer apartment houses, family houses and semi-detached houses etc. Only students aged 14–15 were selected for the analysis (children between 6 and 15 years attend the elementary school in Czechia, thus we used the students of the last grade). Altogether, 407 usable responses were received.

The questionnaires were filled out in class, usually during ICT lessons. The questionnaire design has been scrutinized by experts from various fields including children psychiatry, toxicology, demography, medicine, and human geography. The research progress followed the ethical guidelines proposed by the Czech government, thus all procedures were performed in compliance with relevant laws and institutional guidelines which appropriate institutional committees have approved. The written consent of the school director was arranged beforehand as a necessary condition for carrying out the survey. Students were given a unique code for each school ensuring the anonymity of individual data. After entering this school-code, the on-line survey form opened and could be filled out on their computers. To ensure confidentiality, the recorded and immediately anonymized data were available only to the researchers and the supervisors of this project, protected by password and stored only at the fire-walled servers set at Faculty of Science.

Most of the respondents came from blocks of flats - the typical housing estates built in Prague during the communist era (43.7%), the second largest sample of students lived in newly constructed houses in suburbia (15%), while the other most numerous groups lived in older family houses (12%) and older city apartment houses (11.5%). Newer apartment houses within recently built residence complexes were the residence of 10.3% of respondents, 3.9% of students lived in row houses and 3.4% of students in semi-detached houses.

The following three types of health risk behaviors (HRBs) were assessed.

#### Alcohol consumption

In the questionnaire students were asked: “Think back over the last 30 days. How many times (if any) have you had five or more drinks on one occasion?” Answering options were: “Never” “Number of occasions: 1”, “Number of occasions: 2”, “Number of occasions: 3-5”, “Number of occasions: 6-9”, “Number of occasions: 10 or more”.

Those reporting 5 or more drinks on 3 or more occasions were considered as binge drinkers and coded as cases of health risk behavior (see Table 1 for their distribution across the types of built environments). Social scientists and epidemiologists, use quantitative definitions of binge drinking based on the number of drinks consumed on one occasion. The traditional 5+ HED (heavy episodic drinking) measure is a traditionally used indicator of alcohol-related risk [37].

**Table 1:** **Data set according HRB and neighborhood type**

Neighborhood type	Daily smoking		Binge drinking		Marijuana use		Without HRB		One HRB		Two/three HRB		Sample size
	N	%	N	%	N	%	N	%	N	%	N	%	
Blocks of flats	20	11.2	23	12.9	51	28.7	113	63.5	44	24.7	21	11.8	**178**
New family houses in suburbia	10	16.4	5	8.2	13	21.3	43	70.5	11	18.0	7	11.5	**61**
Row houses	3	18.8	2	12.5	4	25.0	11	68.8	3	18.8	2	12.5	**16**
Older city apartment houses	9	19.1	5	10.6	10	21.3	31	66.0	9	19.1	7	14.9	**47**
Newer apartment houses	5	11.9	4	9.5	10	23.8	29	69.0	9	21.4	4	9.5	**42**
Family houses	7	14.3	6	12.2	20	40.8	27	55.1	12	24.5	10	20.4	**49**
Semi-detached houses	0	0.0	0	0.0	3	21.4	11	78.6	3	21.4	0	0.0	**14**
**Total**	**54**	**13.3**	**45**	**11.1**	**111**	**27.3**	**265**	**65.1**	**91**	**22.4**	**51**	**12.5**	**407**

Bold - bold text shows the significant results within the table.

#### Smoking

“How frequently have you smoked cigarettes during the last 30 days?” Answering options were: “Not at all”, “Less than 1 cigarette per week”, “Less than 1 cigarette per day”, “1–5 cigarettes per day”, “6–10 cigarettes per day”, “11–20 cigarettes per day” and “More than 20 cigarettes per day”. Those reporting smoking ≥1 cigarettes per day were considered as daily smokers - risk tobacco users (see Table 1 for their distribution across the types of built environments).

#### Marijuana use

In the questionnaire students were asked: “On how many occasions (if any) have you used marijuana or hashish (cannabis) during the last 12 months?” Answering options were: “Never”, “1–2”, “3–5”, “6–9”,“10–19”, “20–39” or “40 or more”. Those reporting cannabis use more than 6 times during the previous year were considered as marijuana users and coded as cases for this type of health risk behavior (see Table 1 for their distribution across the types of built environments).

The above measures were used to determine the prevalence of the single HRB among students. Subsequently, subjects were classified as having none, one, two or three types of considered HRB to evaluate the level of comorbidity. The distribution of respondents according the home built environment they live in and their HRB, including its co-occurrence is displayed in the Table 1.

Respondents were also asked for their opinions of their indoor and outdoor home and school built environments. The environment was assessed using a 26-item scale. We evaluated both external (8 items for the school locality and 5 items for the home locality) and internal qualities of home (10 variables – 5 items for the home locality and 5 items for the home building) and school environments (16 variables - 8 items for the school environs and 8 items for the school building). Whereas U.S. studies use severe measures of neighborhood disorder, such as drug dealing, drug use, gang activity, deteriorated buildings, violence, shootings, prostitution, unresponsive police etc., such problems are not typical for the European (or Central European) setting. Thus, we use similar characteristics, but ones that were found appropriate for the Czech context (e.g. neighborhood disorder due to racial, ethnic or religious differences, litter, vandalism, abandoned buildings, neighborhood dilapidation, scarcity of green areas and playgrounds, traffic congestions etc.).

The second important methodological feature of our pilot study is that we chose to let the responding teenagers evaluate their environment according to their own perception of it. Many foreign studies use aggregate data and only a few [38, 39] opt for subjective measures of the social context via the adolescents’ own perceptions of their neighborhood and school environment. As Winstanley et al. [25] show, individual perceptions of neighborhood are as important as external or objective measures in research into adolescent alcohol or drug use. The items in this scale scored from 1 to 4 points, from non-problematic and desirable environments to unpleasant and problematic ones (the evaluation has been coded as 1 = no problem, 2 = small problem, 3 = bigger problem, 4 = serious problem), so that higher scores indicated a greater degree of neighborhood disadvantage.

The date were transferred into a database and analyzed using statistical analysis with the help of the SPSS (Statistical Package for the Social Sciences), version 17 (SPSS, Chicago, IL). Principal component analysis (PCA) was utilized as a form of multidimensional scaling. PCA allowed us to identify underlying variables that explain the pattern of correlations within a set of observed variables and explore the latent structure of the variables in data file. We calculated component loadings and scores. The component scores computed for each participant were used for further mean analyses.

First, descriptive analyses were conducted to explore the nature of the data and their basic distributions across the different environments and risk behaviors. Principal component analysis of the measures evaluating home and school indoor and outdoor environments followed in orderdentify the key factors behind the students’ perceptions of their environs. Third, comparison of component score means was used to identify what built environment types of residence led to particular evaluations of home and school environment.

### Results of the questionnaire survey of Prague’s teenagers

Since any significant variations for gender were not observed, we do not present all the following results separately for girls and boys. As Table 1 shows, binge drinking was reported mainly by students living on housing estates with blocks of flats (12.9%), in row houses (12.5%) and those living in neighborhoods of family houses (12.2%). When it comes to smoking, the most frequent occurrence of daily smokers was found in older city apartment houses (19.1%), in neighborhoods of row houses (18.8%) and residential quarters of new family houses (16.4%). Table 1 shows a high prevalence of risky marijuana use in almost all the surveyed types of neighborhood, with the most severe situation evidenced for older family houses (40.8%) and blocks of flats (28.7%).

Less than two thirds of students did not engage in multiple health-risk behavior, 22.4% reported one risk, 12.5% two or more risks. The best situation was reported for the built environment of semi-detached houses, new family houses and newer apartment houses. The fewest teenagers without any risk behavior were found in older family houses. One fifth of the respondents living in neighborhoods of family houses reported multiple health risk behaviors.

In the analytical section of our paper, we used component analysis to identify underlying factors that explain the pattern of correlations within a set of observed variables characterizing the home (residential) and school environment. After entering the variables evaluating the students’ perception of the home and school environmental aspects we used principal component analysis to exclude two components. As Table 2 shows, the first component (C1) is the most closely correlated with external characteristics of neighborhoods, especially with drugs, racial riots, violence and general dilapidation of neighborhoods. The second component (C2) is the most closely correlated with internal characteristics of built environment, e.g. indoor-air and temperature discomfort.

**Table 2:** **Loadings on the two components of the home environment (outdoor and indoor)**

Variables of the home environment	Component	
	C1	C2
Drugs, alcohol used in the vicinity	**.869**	.136
Violence, vandalism in the neighborhood	**.847**	.193
Neighborhood dilapidation	**.826**	.258
Neglected natural environment and lack of greenery	**.812**	.217
Racial or religious problems	**.754**	
Temperature comfort	.146	**.847**
Indoor air quality	.156	**.840**
Condition of toilets		**.807**
Need for repairs	.180	**.649**
Quality of sport facilities	.199	**.601**

*Note:* the two first principal component loadings from the total number of 10 are in the table above. Bold loadings *-* The variables describing the outdoor environment have the highest loadings for Component 1, the variables describing the indoor environment have the highest loadings for Component 2. The first component is most highly correlated with the variables *Drugs, alcohol used in vicinity* and *Violence, vandalism in the neighborhood*. The second component is most highly correlated with the variable *Temperature comfort*. Missing values are lower than the absolute value of 0.100.

A similar component analysis was also processed for the subjectively evaluated characteristics of the school environment (Table 3). The problematic issues forming the first component were characteristics describing the external school environment, especially the lack of green spaces, violence and vandalism, drugs, and dilapidation of buildings. Component 2 is comprised mainly of characteristics of the internal school environment, such as the condition of sport facilities, air quality and outdoor places for sports.

**Table 3:** **Loadings on the two components of the school environment (outdoor and indoor)**

Variables of the school environment	Component	
	C1	C2
Neglected natural environment and lack of greenery	**.841**	.120
Violence and vandalism in the school environs	**.809**	
Drugs used in the vicinity of school	**.790**	
Run-down or vacant buildings	**.743**	.132
Neighborhood dilapidation	**.701**	.285
Traffic problems	**.678**	.168
Lack of sport and leisure facilities	**.661**	.256
Racial or religious problems	**.650**	
Condition of gym	.130	**.742**
Air quality in school	.143	**.700**
Condition of outdoor sport facilities		**.685**
Wheelchair accessibility		**.661**
Need of repairs	.140	**.652**
Condition of toilets	.183	**.642**
Condition of specialized classrooms	.132	**.616**
Temperature comfort	.143	**.606**

*Note:* the two first principal component loadings from the total number of 16 are in the table above. Bold loadings *-* The variables describing the outdoor environment have the highest loadings for Component 1, the variables describing the indoor environment have the highest loadings for Component 2. The first component is most highly correlated with the variables *Neglected natural environment and lack of greenery* and *Violence and vandalism in the school environs*. The second component is most highly correlated with the variable *Condition of gym*. Missing values are lower than the absolute value of 0.100.

We then analyzed the relation between the perception of the external features of home and school environment and particular neighborhood types (Table 4) by comparing the mean factor scores on the particular outdoor and indoor components for each surveyed type of environment. A higher score on the two indices represents a more serious perception of the environment and higher levels of discomfort. Not surprisingly, the perception of deterioration of the built environment (its external features) was associated with living in blocks of flats within housing estates in Prague. Living in semi-detached houses and suburbia, on the contrary, led respondents to evaluate their neighborhood environments more positively. Living in newer apartment houses, older city apartment houses and semi-detached houses led to a worse assessment of the built environment around schools, which may be explained by the fact that these schools are located in typical inner city neighborhoods of Prague, where the school buildings originate from the beginning of the 20th century with an interior structure typical of that time and there is little larger space for further development of modern school facilities (playgrounds, gardens, sport facilities).

**Table 4:** **Assessment of outdoor features of the home and school built environments by neighborhood types (means of component scores)**

Neighborhood type	Home environment	School environment
Blocks of flats	.197	.010
New family houses in suburbia	-.329	-.153
Row houses	-.167	-.259
Older city apartment houses	-.021	.115
Newer apartment houses	-.056	.165
Family houses	-.052	-.051
Semi-detached houses	-.503	.111

*Note*: Means of relevant outdoor component scores for neighborhood types.

A higher positive score represents a more serious perception of the environment and higher levels of discomfort.

The same analysis was also conducted for the characteristics of the indoor environment (Table 5). In this case, the indoor environment quality was evaluated worst by those living in family houses and semi-detached houses. As regards the school environments, the most problematic perception of the school indoor environment was evidenced for the blocks of flats, which refers to the fact that the students from the housing estates often attend schools within the same environments, also built during the communist era and with similar architectural qualities.

**Table 5:** **Assessment of indoor features of home and school built environment by neighborhood types (means of component scores)**

Neighborhood type	Home environment	School environment
Blocks of flats	.088	.109
New family houses in suburbia	-.160	-.134
Row houses	-.427	-.081
Older city apartment houses	.023	-.166
Newer apartment houses	-.194	.032
Family houses	.109	-.181
Semi-detached houses	.253	.074

*Note*: Means of the relevant indoor component scores for neighborhood types.

A higher positive score represents a more serious perception of the environment and higher levels of discomfort.

Finally, we compared the mean evaluations of responses to the questions evaluating the home and school environment within particular neighborhood types (not shown) to find out which features of the environment are viewed as the most problematic by students coming from different built environments. Higher means represent more problematic perceptions of the features.

Teenagers from the housing estates saw problems mainly in neighborhood dilapidation and the existence of drugs and violence in their neighborhoods. The second most critically evaluated environment is the semi-detached houses, whose residents gave the lowest evaluation to the lack of sport facilities, and to the existence of drugs and violence in their vicinity. The students from older apartment houses tended to a critical evaluation of the indoor quality of their homes which need repairs, as did students from older family houses. The students from new family houses in suburban areas, newer apartment houses and row houses had the best evaluations of their home environments, suggesting that the newly constructed residential complexes and houses have a higher quality of both outdoor and indoor environments and are much better accepted and perceived by the resident teenagers.

The respondents were more critical in their evaluation of school outdoor and indoor characteristics. The highest mean assessment values were reported for teenagers living in neighborhoods with semi-detached houses, whose evaluations were above 2 in almost all the cases. Again, the worst problem seemed to be the accessibility of school, neighborhood disorders and the appearance of drugs in the vicinity of the school. Accessibility, indoor air quality and the cleanliness of toilets were among the most serious problems mentioned by residents of housing estates, while disorder, drugs and violence in the neighborhood also received high marks. The students from older family houses emphasized the accessibility of schools and the indoor air quality and temperature discomfort. Similarly, students from new suburban family houses did not like the accessibility, air quality, temperature and toilets in their schools, and the same is true for students from the neighborhoods of row houses and those living in newer apartment houses. This implies that less attention is paid to schools and their surroundings than to home environments, as even students from better quality neighborhoods assess some features of their school environment critically.

The fact that the evaluation of school environment characteristics is slightly more important for the prediction of health risk behaviors is also revealed in the last analysis (Table 6), where we compared the factor score means for each of the components extracted (home and school outdoor and indoor) for risk behavior occurrence and its comorbidity. A problematic school built environment is also associated very significantly with the co-occurrence of multiple health risk behaviors (Table 6) – in the case of two and more risk behaviors, the school outdoor characteristic seem to have the highest formative role. Home environment outdoor features are also very important for the emergence of both one and more health risk behaviors, while the relation of home indoor characteristics is positive, although slightly weaker.

**Table 6:** **Assessment of out/indoor school and home built environments and HRB’s (means of component scores)**

Comorbidity	School environment outdoor characteristics	School environment indoor characteristics	Home environment outdoor characteristics	Home environment indoor characteristics
Without HRB	-.072	-.129	-.194	-.074
One HRB	-.091	.**350**	**.288**	.085
Two and three HRB	**.535**	.082	**.414**	.204

*Note:* Means of the relevant component scores of HRB’s.

A Higher positive score represents a more serious perception of the environment and higher levels of discomfort.

Bold - Higher scores.

The school environment has been perceived more critically than that of home, showing a significant degree of dissatisfaction with the school milieu among the surveyed students in Prague. Moreover, it also proved to be a stronger predictor of health risk behavior and its co-occurrence (Table 6). Considering the lifestyle theory, emphasizing the role of the environment where the individual spends most of their time out of the home, and opportunity theories in general, we next shifted our attention to the school environs and the schools themselves in our study. Our choice of school environments for detailed field research was supported by the fact that schools are often mentioned among the so-called crime generators, the facilities or buildings that bring large numbers of diverse people together [40], which in our case of teenagers represents another potentially strong risk factor. The presence of more teenagers increases the anonymity of the place and results in an ignoring of guardianship activities [11].

### Data and methods: field research into the characteristics of the school environment

Despite the fact that many U.S. studies confirm the dominance of social sources for obtaining alcohol, tobacco, and other drugs above commercial sources [41], the commercial access mainly to alcohol and tobacco of underage teenagers is a serious issue. This is true, especially as the age of the teenagers increases, but still remains under the legislative minimum [42]. What is more, Czechia has the highest perceived availability of cannabis, alcohol and cigarettes [32], low law enforcement in general (when it comes to the legislative banning of the use of alcohol, smoking or illicit drug consumption), and almost non existing community policies preventing alcohol or tobacco use by underage users. The choice to study the “opportunity hot spots” around particular schools therefore seemed to be an appropriate research direction.

Each of the 19 elementary schools and their environments that were selected for the questionnaire survey in the first phase of the research were visited by a trained researcher. A circle of approximately 250 m around the school (distorted to contain whole blocks of houses) had been previously identified on a map using GIS to delimitate the area the researcher had to visit and to thoroughly check for any potential opportunity hot spots – liquor stores, small convenience stores, gambling clubs, restaurants, sales booths, newsstands etc. These opportunity hot spots were recorded on the map and photographs of these spots were taken. The school itself was also checked, its building, facilities and environs were photographed and a short description of the environment characteristics was provided in the form of field research notes. To supplement the field information, each school website was visited to view the principal school documents which contain any reference to the prevention of risk behavior.

### Results of the field research into the characteristics of the school environment – the local context

The previous results of quantitative analysis show that home outdoor environment characteristics were worst evaluated by students from housing estates with blocks of flats and the home indoor environment, quite surprisingly, by the students from semi-detached houses and family houses (which may be caused by the greater age of these buildings and thus lower perceived quality of the interiors). Since the field research clearly focused on schools and their environments, the previous analysis directed our attention mainly towards older and newer city apartment houses where the evaluation of the school outdoor environment had been poor and to housing estates with blocks of flats where it was school indoor characteristics that were criticized (neighborhoods of semi-detached houses are not described in the further analysis due to the low number of students residing in these types of built environment).

**Figure 1:**
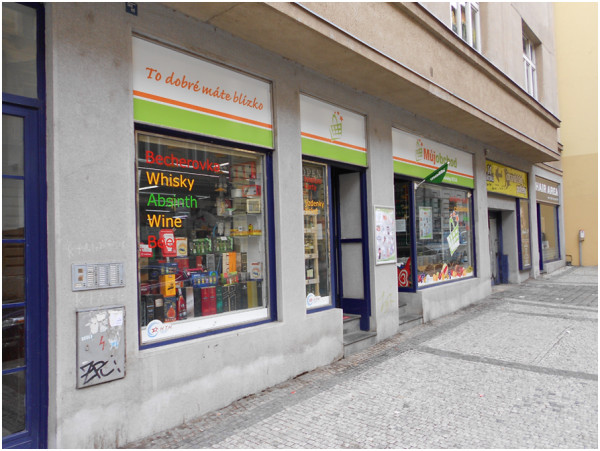
**A typical small convenience store in the inner city of Prague.** Note: This shop is located across the street from one of the elementary schools included in the survey. The title of the shop says “to dobré máte nablízko” which means “good things are at hand”, but the offer on the shop window speaks for itself - Becherovka (a typical Czech herb liquor), Whisky, Absinth, wine and beer are promoted as the “key” items on sale. Source: authors.

Whereas the schools in the neighborhoods of recently constructed apartment houses did not demonstrate an alarming number of opportunity hot spots or other critical places, those schools in neighborhoods with older apartment houses (typical historical centre and inner city neighborhoods) were definitely the environments with the highest density of opportunity hot spots. Five schools located in these environments had an average of 41 hotspots in their surroundings (compared to the average of only 7 hot spots in or around the suburban schools, which were evaluated as the best school outdoor environments). It is no surprise that the older apartment houses neighborhoods represent the worst school environments for the surveyed teenagers, as these are neighborhoods with a dense built environment and thus a neglected natural environment and lack of public greenery, signs of vandalism around the school buildings, rundown and dilapidated buildings in general, and many traffic problems. These neighborhoods do not provide a healthy space for children and teenagers to grow up, but on the contrary are an ideal location for restaurants, bars, clubs, liquor shops and small convenience stores (Figure 1), since the population density is high and tourists often also visit and use these neighborhoods.

**Figure 2:**
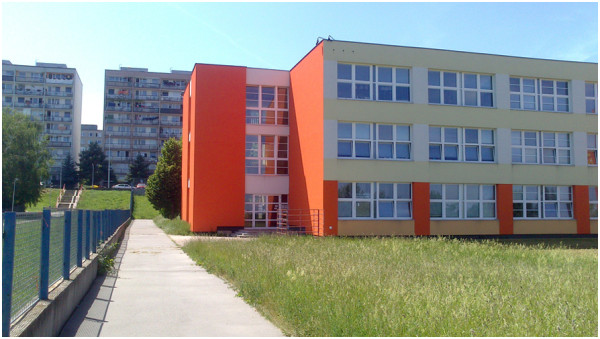
**A typical elementary school within the typical neighborhood of housing estates.** Note: The picture depicts a typical elementary school wedged into the fabric of a housing estate from the 1980s. Although the school building has been renovated, it still does not represent a truly inspiring environment for learning and play. Source: authors.

The housing estates represent another interesting category, being the environment where students felt the worst quality of indoor school environments. This may be due to the fact that the schools within the typical housing estates were built during the construction of these areas, thus in the 1970s and 1980s). Despite the fact that they are undergoing some renovation and reconstruction, they apparently do not represent a truly stimulating environment for study (Figure 2).

The same applies where the environment of the teenagers’ homes were housing estates with blocks of flats. Despite the fact that these environments did not provide high numbers of opportunity hotspots (on average 8 hotspots for the surveyed housing estate locations), they do not possess many qualities of healthy environments when it comes to drugs or alcohol used in the vicinity of the houses, violence and vandalism in the neighborhoods, general dilapidation, neglected greenery and some racial problems as well.

## Discussion

Our analysis shows that different risk behaviors may evolve in different neighborhoods. Alcohol misuse was more frequent within rather “older” dated residential environments - in blocks of flats, row houses or older family houses. This is most likely caused by the fact that in the housing estate built environment, social control tends to be low. As various studies (e.g. ESPAD 2011) show, alcohol is easily accessible to the majority of teenagers and since it is truly inexpensive, it is no surprise that it will be widely consumed in an environment where the young people see enough examples of drinking behavior, both in public or at home [43]. Strategies to ameliorate alcohol use by teenagers often focus on reducing or eliminating the primary sources of alcohol [44] largely targeting commercial access [45]. However, this may be a tricky strategy in the case of Prague, as it was found that there is not a significantly higher density of alcohol outlets on housing estates. Moreover, as many studies have evidenced, parents, guardians or friends are by far the most common sources of alcohol [42] and these sources would not be affected by any strategy aimed at law enforcement or control of possession by underage drinkers [44].

Smoking was, on the other hand, found in the older city apartment houses, row houses and new family houses in suburbia. Cigarettes are an item where the price is rising and so their consumption requires more resources. It can be expected that teenagers in higher income neighborhoods such as in suburbia or in new residential row houses may have more disposable income to buy these [46]. In this case, it would be interesting to push for stronger interventions with merchants. As Landrine et al. [47] claim, asking about age or requiring identification document decreases sales of cigarettes to minors.

The situation with marijuana use showed unexpectedly high results across all the neighborhood types, documenting the formerly revealed high prevalence of marijuana use in Czechia and its big cities [34]. Such high use of marijuana is striking also when compared to the rest of the Central European countries. Czechia is a transit country and many drugs are transferred through the country on their way to the Western Europe. Availability of marijuana is thus widespread and the prices are obviously accessible even for younger customers. While the proportion of those who never tried marijuana in their lifetime is decreasing, there is a clear increase in the categories of students who experiment with drugs or use them regularly. This Czech phenomenon requires further study as regards the sources of offer, but also the causes of demand for marijuana among Czech teenagers. Reducing both access to substances and the motivation to use them is a generally accepted effort to prevent drug use [41].

The quality of environment one lives in and the quality of school environs the teenagers daily attend has a strong relation to the fact of whether an individual becomes involved in particular types of health risk behaviors or even in a combination of these. In our research, the school environment (especially its outdoor features) proved to have a slightly stronger influence mainly on the occurrence of multiple risk behaviors.

The type of built environment where individuals dwell was also perceived differently by the students. In our study, those environments constructed relatively recently, which are more cared for and have not had time to become dilapidated, are far better evaluated by the students than those constructed earlier, such as traditional self-contained family houses, semi-detached houses in traditional outer city zones and older city apartment houses, especially those in the city parts which have not experienced waves of gentrification. This finding clearly illustrates the importance of urban renewal projects focusing on physical, social and economic improvements within disadvantaged neighborhoods. The effect of such improvements on health, health inequalities and quality of life is known from elsewhere (e.g. the Barcelona study [48]). It is thus clear that renovations cannot consist of physical improvement of houses alone, but have to embrace a much wider spectrum of interventions within the dilapidated neighborhoods and disadvantaged communities.

## Conclusion

It is clear that the quality of the environment people live in and individual perceptions of this may influence the health of individuals. However, the relation between environmental characteristics and risk behavior is complex and complicated. Our pilot study pointed to the fact that school environments are evaluated much more critically by teenagers than their home environments. Since school is the place where teenagers spend a great part of their time, the quality of the school environment has to be given more attention by local government managing the public space around and close to the school buildings.

Internationally significant novelty of this paper can be seen in the fact that this paper presents the first study of the phenomenon of risk behavior in neighborhood environment context in post-communist countries. Contrary to the widely accepted knowledge, it shows that it is not the environment of typical communist housing estates which stresses Prague’s teenagers most when it comes to school environs, nor do the housing estates provide more opportunities to access alcohol or tobacco. The most problematic neighborhoods are those in the historical center or inner city with older apartment houses, where the density of diverse people and commercial activities is greater and commercial interests limit the possibilities of public health policies, prevention and protection of young people. This fact is true also when we step out of Prague to the other cities of Czechia (and post-communist countries in general).

The housing estates often represent a kind of social housing in developed countries (USA, Western Europe), but in the post-communist countries case they are characterized by a much more diverse social structure. The same is true for the possession of a family house, which is not necessarily proof of a higher standard of living. Similarly, those dwelling in the new suburban areas come from different social strata and have varying social status. Although the role of the home environment, the quality of life in the indoor and outdoor aspects maintained in and around the teenager’s house are important, parental example, family background and community are extremely important as well. Since our study did not verify a clear link between the built environment and risk behaviors, it must be at the community and family level that the future research agenda has to focus on.

## Authors’ information

JS received her Ph.D. in Social Geography from Charles University in Prague and is an associate professor at the Department of Social Geography and Regional Development, Charles University, Prague. Her research interests include health and behavior, lifestyles and sustainable development. DD is the Head of the Department of Social Geography and Regional Development and focuses on the statistical data analysis in medical geography. MP is a postgraduate student at the Department of Social Geography and Regional Development.
